# Impact of Metal-Based Nanoparticles on Cambisol Microbial Functionality, Enzyme Activity, and Plant Growth

**DOI:** 10.3390/plants10102080

**Published:** 2021-09-30

**Authors:** Sergey Kolesnikov, Alena Timoshenko, Tatiana Minnikova, Natalia Tsepina, Kamil Kazeev, Yulia Akimenko, Alexander Zhadobin, Victoria Shuvaeva, Vishnu D. Rajput, Saglara Mandzhieva, Svetlana Sushkova, Tatiana Minkina, Tamara Dudnikova, Mahmoud Mazarji, Saud Alamri, Manzer H. Siddiqui, Rupesh Kumar Singh

**Affiliations:** 1 Academy of Biology and Biotechnology Named D.I. Ivanovsky, Southern Federal University, 344090 Rostov-on-Don, Russia; kolesnikov1970@list.ru (S.K.); aly9215@mail.ru (A.T.); loko261008@yandex.ru (T.M.); cepinanatalia@yandex.ru (N.T.); kamil_kazeev@mail.ru (K.K.); akimenkojuliya@mail.ru (Y.A.); v_shuvaeva@mail.ru (V.S.); msaglara@mail.ru (S.M.); terra_rossa@mail.ru (S.S.); tminkina@mail.ru (T.M.); tyto98@yandex.ru (T.D.); mahmoudmazarji@gmail.com (M.M.); 2 Rostov on Don Zoo, 344039 Rostov-on-Don, Russia; a90981@yandex.ru; 3 Department of Botany and Microbiology, College of Science, King Saud University, Riyadh 11451, Saudi Arabia; saualamri@ksu.edu.sa (S.A.); mhsiddiqui@ksu.edu.sa (M.H.S.); 4 Centro de Química de Vila Real, Universidade de Trás-os-Montes e Alto Douro, Quinta de Prados, 5000-801 Vila Real, Portugal; rupeshbio702@gmail.com

**Keywords:** ecotoxicity, Cambisols, biotesting, stability, degree of sensitivity, informative value

## Abstract

An increase in the penetration of metal-based nanoparticles (NPs) into the environment requires an assessment of their ecotoxicity as they impair the critical activity of plants, animals, bacteria, and enzymes. Therefore, the study aimed to observe the effects of metal-based NPs, including copper (Cu), nickel (Ni), and zinc (Zn), on the Cambisols, which cover a significant part of the earth’s soil and play an important role in the biosphere. Metal-based NPs were introduced into the soil at concentrations of 100, 1000, and 10,000 mg/kg. The biological properties of the soil are being investigated as the most sensitive to external contamination. The highest ecotoxicity of the studied pollutants introduced into the soil at the same concentrations was shown by Cu (up to 34%) and Zn (up to 30%) NPs, while Ni NPs showed less (up to 22%). Microbiological (total number of bacteria, *Azotobacter* sp. abundance) and phytotoxic properties (radish seed germination and length of roots) of Cambisols were more sensitive (22–53%) to pollution by NPs of Cu, Zn, and Ni, while enzymatic activity (catalase and dehydrogenases) showed less sensitivity (14–32%). The present results could be useful for biomonitoring the state of contaminated soils, especially by NPs.

## 1. Introduction

Cambisols occupy a significant proportion of the earth’s soil cover. They grow a large number of agricultural products [1,2]. Therefore, it is important to study the negative consequences of the contamination of these soils with metal-based nanoparticles (NPs). In recent years, there has been much tension around the issue of contamination of environmental objects by various chemicals, including metal-based NPs. For the time being, NPs are widely used in many fields of science and technology [3]. Nanoparticles are used in the production of thousands of industrial plants, thus it is impossible to stop their accumulation in the environment. Nanotechnology remains one of the most demanded directions in the development of science and technology all over the world. In recent years, nanotechnology has moved from a revolutionary technology to a development tool and is used in many industries, including new materials, electronics, pharmaceuticals, and energy. In terms of public investment in the nanotechnology industry, the US are recognized as the top investor with more than $ 1.8 billion in 2020. In total, the US federal government has financed about $ 31.0 billion in cumulative research and development in the field of nanotechnology so far [4].

According to the forecasts of analytical agencies, the global nanotechnology market continues to develop. Its annual growth rate will average 12.9% for the period from 2020 to 2027 [5]. The main drivers of the nanotechnology market will be applications in medicine and healthcare, ecology, and renewable energy sources. The unique properties of NPs make their impact on human and environmental health and safety hardly predictable. Over the past five years, the penetration of NPs into the soil system from various sources and their effect on soil organisms have been studied [6,7,8,9]. The properties and structure of the soil play an important role in assessing the effect of NPs on soil organisms [10]. Since nanotechnology is an area of innovation and research growth with exponential production, additional information on the impact of NPs on the environment and, in particular, on the soil is required [11].

The analysis of the studies already carried out demonstrated the ambiguity of the results obtained: some authors indicate the absence of a negative impact of NPs entering the environment, while others note a considerable negative impact of the interaction of NPs with environmental components [12,13,14]. A negative impact on the number and activity of bacteria in the soil was recorded under contamination with CuNPs [15,16], ZnNPs [13,17,18], and NiNPs [15]; on the state of plants when contaminated with CuNPs [19,20,21,22], NiNPs [15,23,24], and ZnNPs [25,26,27] and on enzyme activity in soil upon contamination with CuNPs [28,29,30], ZnNPs [29], and NiNPs [15,31]. However, a number of studies have noted the stimulating effect of ZnNPs and CuNPs on the enzymatic activity of soils [32,33,34] and the state of plants [35,36,37,38,39,40].

The available studies on the toxicity of NPs are of local nature and are aimed at studying any one component of the environment. A comprehensive study of biological indicators is required to form a more complete understanding of the consequences of pollution by NPs of the environment. Thus, toxicity studies of NPs are at the beginning, and more comprehensive studies for analyzing the impact of NPs on environmental components and revealing their toxicity mechanisms are required [41,42].

Copper, Zn, and Ni NPs were selected for the study since they are the main pollutants among the manufactured NPs. Copper NPs are widely used in catalysts, gas sensors, heat transfer fluids, microelectronics, and cosmetics [43]. Zinc NPs are found in skin and hair care products, sunscreens, coatings, ceramics, and paints [44]. Nickel NPs are widely used in industry as printing inks, ceramics, and catalysts, as well as in the electrical and electronic industries, and exhibit cytotoxicity against cancer cells [45]. Therefore, they have received particular interest in biomedicine and agriculture [12,18,23,41].

To assess the state of soil after pollution, it is advisable to use indicators of the biological state of soils, such as the activity of soil enzymes, changes in phytotoxic indicators, and the number of soil bacteria [9,13,15,30,31]. Thanks to these biological indicators, it is possible to assess the state of the soil after and during pollution, as well as to assess the rate of restoration of soil health.

The study aimed to investigate the impact of metal-based NPs, i.e., Cu, Ni, and Zn on Cambisol microbial functionality, enzyme activity, and plant growth.

## 2. Results

### 2.1. Influence of CuNPs, NiNPs, and ZnNPs on Microbiological Indicators of Cambisols

**Figure 1:**
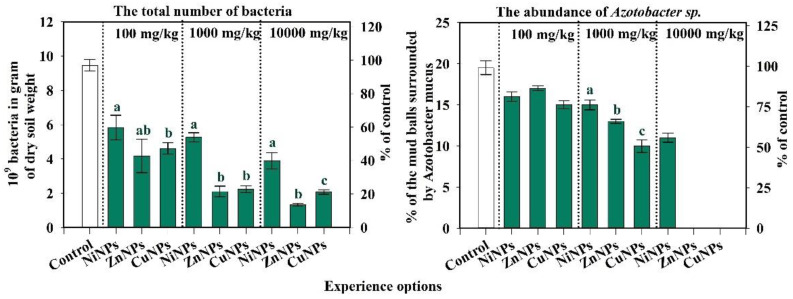
Changes in the total number of bacteria and *Azotobacter* sp. abundance of Cambisols by Cu, Ni, and ZnNPs pollution. Note: Different letters indicate significant differences (*p* < 0.05) between the content of nickel, zinc, and copper nanoparticles at the same dose of their introduction into the soil, obtained as a result of the Student’s test. The absence of bands in the graph indicates the non-viability of the abundance of *Azotobacter* sp. with the introduction of 10,000 mg/kg of zinc and copper nanoparticles into the soil.

The study found an inhibiting effect of CuNPs, NiNPs, and ZnNPs on the total number of bacteria (Figure 1). A statistically significant difference was found between the total number of bacteria and *Azotobacter* sp. abundance between the soil of the control variant and NPs contaminated with Ni, Zn, and Cu in different doses. The smallest effect on the total number of soil microorganisms has been observed by the introduction of NiNPs into the soil. In the variants of the experiment with the introduction of ZnNPs and CuNPs into the soil, the number of bacteria is significantly lower than with the introduction of NiNPs (at *p*-level < 0.05). The greatest influence was exerted by ZnNPs. At a 100-mg/kg concentration, a decrease in this indicator was observed when contaminated with ZnNPs, CuNPs, NiNPs—56, 48, and 39% of the control, respectively. It was also observed that, with an increase in the concentration of pollutants, the toxic effect increases. 

With the introduction of pollutants into the soil at a dose of 10,000 mg/kg, ZnNPs had the greatest effect on the abundance of bacteria since the number of microorganisms in 1 g of soil is much lower than in the variants with the introduction of CuNPs and NiNPs. The *Azotobacter* sp. abundance after pollution by CuNPs, NiNPs, and ZnNPs was also negatively affected. Already, in the lowest studied concentration of 100 mg/kg, a noticeable decrease in this indicator was observed for CuNPs, NiNPs, and ZnNPs, by 23, 18, and 13%, respectively. Differences between the exposures to pollutants can be traced as starting from a dose of 1000 mg/kg. With the introduction of 1000 mg/kg CuNPs, NiNPs, and ZnNPs, the abundance of *Azotobacter* sp. abundance decreases by 49, 23, and 33%, respectively. When 10,000 mg/kg NiNPs were added to the soil, the *Azotobacter* sp. abundance decreased by 46%, and CuNPs and ZnNPs were suppressed by 100%. It was revealed that under the condition of 10,000 mg/kg CuNPs or ZnNPs, *Azotobacter* sp. abundance was not viable (Figure 1).

Thus, the microbiological indicators of the Cambisols were found to be sensitive to contamination with CuNPs, NiNPs, and ZnNPs. According to the degree of influence of NPs on the total number of bacteria of Cambisols, the studied metals the following series was formed: Zn > Cu > Ni. According to the degree of influence of NPs on the *Azotobacter* sp. abundance of Cambisols the following series was formed: Cu > Zn > Ni.

### 2.2. Influence of CuNPs, NiNPs, and ZnNPs on the Activity of Enzymes of Cambisols

**Figure 2:**
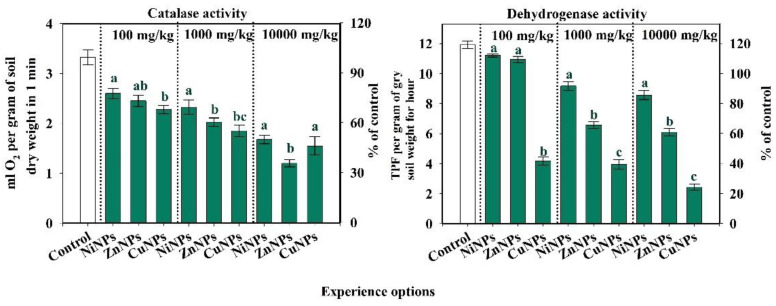
Changes in catalase and dehydrogenase activity of Cambisols by CuNPs, NiNPs, and ZnNPs pollution. Note: Different letters indicate significant differences (*p* < 0.05) between the content of nickel, zinc, and copper nanoparticles at the same dose of their introduction into the soil, obtained as a result of the Student’s test.

The degree of influence of different concentrations of CuNPs, NiNPs, and ZnNPs on catalase activity is shown in Figure 2. The effect of pollutants on enzyme activity is not the same. With the introduction of NiNPs, the catalase and dehydrogenases activity is 95% more likely than with soil contamination with CuNPs or ZnNPs. At the same time, the inhibition of catalase activity takes place to the greatest extent under the influence of ZnNPs in the variants of the experiment with the introduction of 100 and 1000 mg/kg, and CuNPs—10,000 mg/kg. For dehydrogenases, the effect of NPs depends on the types of metal. When ZnNPs are added, the enzyme activity is significantly reduced in comparison with NiNPs, and the CuNPs addition leads to a significant decrease compared to ZnNPs (at *p*-level < 0.05) (Figure 2). Copper NPs and ZnNPs had a similar effect on each other of enzyme activity. At a concentration of 100 mg/kg in soil, CuNPs turned out to be the most toxic with a decrease in catalase activity by 33% from control, while the catalase activity in soil with NiNPs and ZnNPs was decreased by 21 and 24% of control, respectively. However, when the highest dose was introduced into the soil (10,000 mg/kg), it inhibited the enzyme activity after pollution by CuNPs, NiNPs, and ZnNPs by 52, 48, and 64% of the control, respectively. At that concentration, ZnNPs are more toxic than CuNPs.

Copper NPs, NiNPs, and ZnNPs had a negative effect on dehydrogenases activity (Figure 2). The most strongly influenced CuNPs decrease in the indicator by 65, 67, and 80% of control at concentrations of 100, 1000, and 10,000 mg/kg, respectively. The NiNPs had the least effect on the activity of dehydrogenases: a decrease by 23 and 28% of control, respectively (at concentrations of 1000 and 10,000 mg/kg). For all investigated NPs: the higher the concentration was, the more sensitive was this indicator to pollution.

The enzymatic activity of Cambisols is sensitive to contamination with CuNPs, NiNPs, and ZnNPs. According to the degree of influence on the activity of catalase, the metals formed the following series: Cu = Zn > Ni. According to the degree of influence on the activity of dehydrogenases, the metals formed the following series: Cu > Zn > Ni. At the same time, for CuNPs, the activity of dehydrogenases was more sensitive to pollution than the activity of catalase. In contrast, for NiNPs and ZnNPs, the activity of catalase was more inhibited.

### 2.3. Influence of CuNPs, NiNPs, and ZnNPs on Radish Germination and Root Length of Cambisol

**Figure 3:**
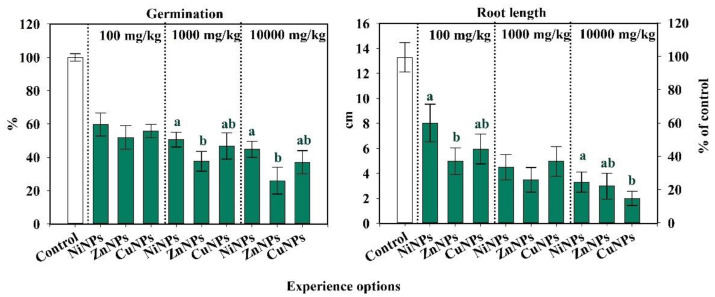
Change in germination rate and root length of Cambisols by CuNPs, NiNPs, and ZnNPs pollution, % of control. Note: Different letters indicate significant differences (*p* < 0.05) between the content of nickel, zinc, and copper nanoparticles at the same dose of their introduction into the soil, obtained as a result of the Student’s test.

The germination rate in Cambisol was most influenced by ZnNPs, and the least by NiNPs (Figure 3). At a concentration of 100 mg/kg, there was a decrease in the germination of radish when contaminated with CuNPs, NiNPs, and ZnNPs by 42, 37, and 47% of the control. However, significant differences in plant germination between the effects of 100 mg/kg NiNPs, ZnNPs, and CuNPs were not revealed (at *p*-level < 0.05). With an increase in the concentration of pollutants, germination was further decreased. When applying 1000 mg/kg CuNPs, NiNPs, and ZnNPs, germination decreased by 53, 47, and 63%, respectively.

Nanoparticles of Cu, Ni, and Zn negatively affected the root length of radish (Figure 3). The ZnNPs had the strongest effect with a decrease of 62, 74, and 77% at concentrations of 100, 1000, and 10,000 mg/kg, respectively. In contrast, NiNPs had the smallest effect on root length of radish, with a decrease of 40, 66, and 75% at concentrations of 100, 1000, and 10,000 mg/kg, respectively. The CuNPs reduced root length of radish by 55, 62, and 85% respectively, at concentrations of 100, 1000, and 10,000 mg/kg. The higher the concentration was, the more sensitive this indicator was to pollution. 

Differences in germination rate and root length after the addition of NiNPs, ZnNPs, or CuNPs were less obvious than other studied parameters (Figure 3).

Thus, the phytotoxicity indices of Cambisols are sensitive to contamination with CuNPs, NiNPs, and ZnNPs. According to the degree of influence on the germination rate and the length of radish roots, the metals formed the following rank: Zn > Cu > Ni. At the same time, the germination rate was more sensitive to pollution than the root length of the radish.

### 2.4. Assessment of the Relationship between Biological Parameters and the Dose of Nanoparticles

**Figure 4:**
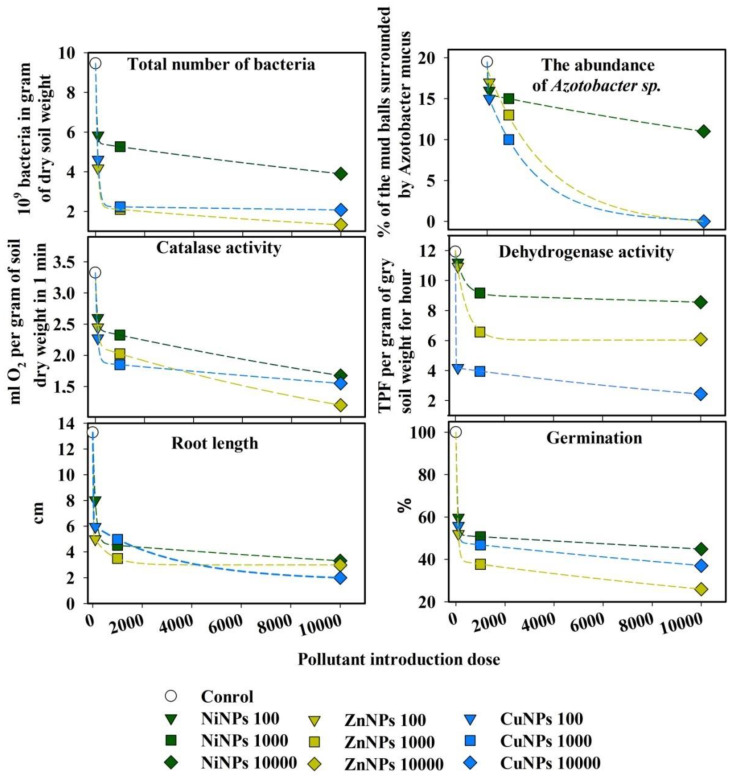
The relationship between the suppression of the microbiological and enzyme indicators of the Cambisols, germination of seeds, and the application rates of various NPs.

Based on the results obtained, a nonlinear relationship was revealed between the dose of various pollutants and the inhibition of microbiological indicators, as well as the germination capacity and length of radish roots (Figure 4). With an increase in the concentration of NPs in the soil, a sharp exponential decline in these indicators was observed. The equations were obtained at *p*-level < 0.05 and had a coefficient of determination in the range from 0.97 to 0.99, which shows a high degree of relationship between the level of pollution and the biological response.

### 2.5. Integrated Index of the Biological State (IIBS) of Cambisols Contaminated by CuNPs, NiNPs, and ZnNPs

**Figure 5:**
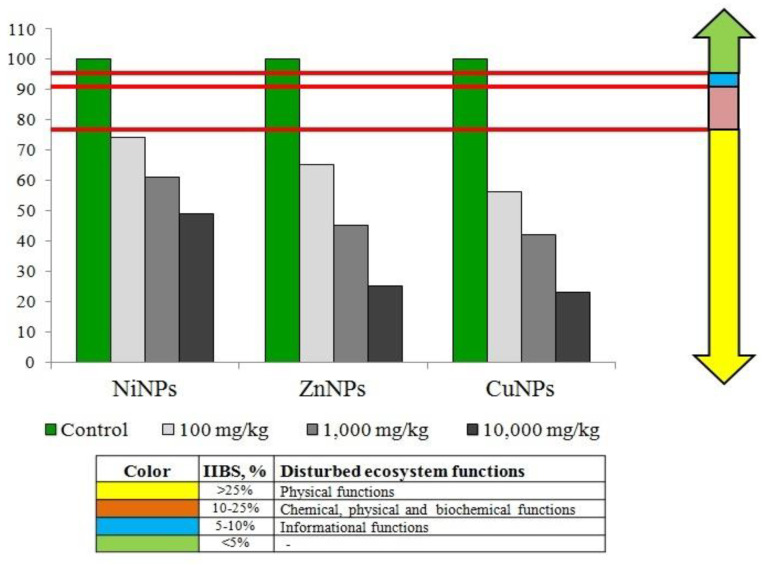
Change in IIBS of Cambisols by Cu, Ni, and Zn NPs pollution, % of control.

The results of calculating IIBS according to the analysis of the influence of CuNPs, NiNPs, and Zn NPs on the state of Cambisols are presented in Figure 5. Although ZnNPs had a stronger effect on some parameters than CuNPs, according to the results of IIBS, CuNPs turned out to be more toxic to Cambisols than ZnNPs. In this regard, metals were found to form the following order: Cu > Zn > Ni. The test substances had the greatest effect at a concentration of 10,000 mg/kg. There was a direct relationship between the concentration of NPs and the degree of deterioration of the biological properties of Cambisols.

## 3. Discussion

Cambisol contamination with CuNPs, NiNPs, and ZnNPs leads to a decrease of microbiological parameters of total number of bacteria and of *Azotobacter* sp. abundance. The CuNPs and ZnNPs appeared to be more toxic for the microbiological parameters of Cambisols’ state than NiNPs. The total number of bacteria was mostly more sensitive to contamination than *Azotobacter* sp. abundance. Concentrations of CuNPs and ZnNPs of 10,000 mg/mg were totally inhibitory for *Azotobacter* sp. abundance. This high concentration of metal-based NPs is very toxic for biological indicators. The *Azotobacter* sp. abundance remained much less sensitive to contamination with NiNPs. The negative impact of CuNPs, NiNPs, and ZnNPs on the enzymatic activity of Cambisols was recorded. The dehydrogenase activity was decreased a little less than the catalase activity. Other researchers also indicate the negative impact of metal-based NPs on the soil enzymatic activity [33,34]. The resistance of Chernozems to pollution by NPs of metal oxides, including CuNPs, NiNPs, and Zn NPs, was shown earlier [15]. Moreover, CuNPs, NiNPs, and ZnNPs had a considerable impact on Cambisols’ phytotoxical properties (germination rate and radish root length). The CuNPs and ZnNPs had the highest approximately equal impact. As seen in Figure 5, at contamination with NiNPs, these indicators’ lowest degree of reduction was observed. The direct dependence of the change of phytotoxic indicators on the concentration of contaminants was observed: the higher the dose, the lower the germination rate as well as radish root length. Researchers also noted the negative impact of metal-based NPs on the state of plants [12,13,14,41]. 

To reveal common patterns of the impact of CuNPs, NiNPs, and ZnNPs, the soil IIBS was calculated. Copper and ZnNPs exhibited the largest inhibited impact on IIBS of Cambisols, but NiNPs hindered the least significant effect on the IIBS of Cambisols. Previous studies have also shown the least effect of Ni NPs among other investigated substances [15,46] and a strong toxic effect of CuNPs on the biological parameters of soils [22,47,48,49]. In addition, our studies have confirmed the hypothesis that the higher the dose of the pollutant, the stronger the inhibition of biological indicators, which is agreement with previously reported results [19,46,50,51].

The information content and sensitivity of the studied indicators were assessed (Figure 1, Figure 2 and Figure 3). The degree of the information content of the indicator was determined by the tightness of the correlation (R) between the biological indicator and the doses of the CuNPs, NiNPs, and ZnNPs in the Cambisols. According to the information value of content, the studied biological indicators were arranged as the following series.

NiNPs:

*Azotobacter sp*. abundance > activity of catalase> activity of dehydrogenases> total number of bacteria ≥ length of roots> germination rate.

ZnNPs: 

*Azotobacter sp*. abundance > activity of catalase > activity of dehydrogenases> germination rate ≥ total number of bacteria > length of roots.

CuNPs:

*Azotobacter sp*. abundance > length of roots ≥ catalase activity ≥ germination rate ≥ total number of bacteria ≥ dehydrogenases activity. 

It emerges from the above list that *Azotobacter* sp. abundance was the most useful in this investigation, as it is the only measureable variable. Other variables are less usable as they are inapplicable due to the fact that they are not detectable, being infeasible to measure, or both of these reasons.

The information content and sensitivity of the studied biological indicators were assessed. The series of the degree of sensitivity to contamination with CuNPs, NiNPs, and ZnNPs of biological indicators of Cambisols can be expressed as follows.

NiNPs:

length of roots ≥ total number of bacteria ≥ germination rate > catalase activity > *Azotobacter* sp. abundance > activity of dehydrogenases.

ZnNPs: 

total number of bacteria ≥ length of roots > germination rate > *Azotobacter* sp. abundance ≥ catalase activity > activity of dehydrogenases.

CuNPs: 

activity of dehydrogenases ≥ total number of bacteria ≥ length of roots> *Azotobacter* sp. abundance > germination rate > activity of catalase.

Thus, microbiological and phytotoxic indicators were the most sensitive to contamination with CuNPs, NiNPs, and ZnNPs. Enzymatic activity indicators were less sensitive. A similar pattern was observed earlier for oxides and water-soluble salts of heavy metal [52]. According to the degree of the negative influence of NPs on the biological indicators of Cambisols, the studied metals formed the sequence: Cu ≥ Zn > Ni. The CuNPs and ZnNPs exhibited greater ecotoxicity than NiNPs. This does not support the existing hypothesis that the toxicity of NPs depends on their size and does not depend on the chemical nature of the element.

By the degree of the negative impact of NPs on the biological properties of Cambisols, the studied metals formed the sequence: Cu ≥ Zn > Ni. The direct dependence of the worsening of soil biological properties on the concentration of metal-based NPs has been observed. No stimulating effects of CuNPs, NiNPs, and ZnNPs was revealed, which frequently occurs at soil contamination with heavy metals. Microbiological and phytotoxic indicators of Cambisols were decreased to the largest degree and the enzymatic activity was less sensitive to contamination with CuNPs, NiNPs, and ZnNPs.

## 4. Materials and Methods

### 4.1. Study Site

The soil for the study was classified as Cambisols according to World Research Base (2015). This type of soil is widely spread throughout Russia and the world [53,54]. The study site (beech-hornbeam forest) was located near the village of Nickel Plant (Russia, the Republic of Adygea, Maykop District, 44°10′39.76″ N, 40°9′27.47″ W). The soil type was characterized as a heavy loam granulometric composition, an average organic matter content of 1.8% and pH_H2O_ = 5.8 (acidic). This experiment sampled Cambisols (soil layer of 0–20 sm.) since heavy metals were deposited at this site [55]. Matal-based NPs have high mobility and are able to exhibit high toxicity in relation to soil biota and plants [9,46].

### 4.2. Experimental Details

The objective of this study was to provide a comprehensive assessment of the influence of CuNPs, NiNPs, and ZnNPs on the enzymatic activity of the soil, microbial, and phytotoxicity indicators based on the concentration of Cu, Ni, and Zn. In this work, NPs of the following sizes were studied: NiNPs, 70–80 nm, ZnNPs, 90–150 nm, and CuNPs, 50 nm. The nanoparticles were provided by the company Advanced Powder Technologies LLC (Russia, Tomsk).

The selected metal-based NPs (Cu, Ni, and Zn: concentrations: 100, 1000, and 10,000 mg/kg) were introduced into the Cambisols (1 kg) in the form of a dry, finely dispersed powder. The NPs were initially mixed with a small amount of Cambisols for uniform distribution and then added to the total Cambisols mass, and poured into the vegetative pots, and incubated under temperature (22–25 °C) in the laboratory. The soil moisture was maintained at 60%. The influence of NPs on the biological properties of Cambisols in the model experiment was assessed 10 days after contamination. A longer incubation period increases the difference in the state of the soil incubated in the laboratory from its state in natural conditions [50].

### 4.3. Measurement Procedures for Biological Indicators

Laboratory studies of biological indicators were performed using the methods indicated (Table 1). It was efficient to use sensitive biological indicators to diagnose soil conditions after chemical pollution [15,46,50,51]. The set of indicators gave an informative estimate of the biological processes taking place in the soil and used the ecological state of the ground. The total number of bacteria, the *Azotobacter* sp. abundance, the activity of catalase and dehydrogenases, and the phytotoxic properties of the soils (germination rate and length of roots of radish (*Raphanus sativus* L.)) were investigated.

#### 4.3.1. Measurement of Cambisols’ Organic Matter and pH

In Cambisols, before contamination of NiNPs, ZnNPs, CuNPs, organic matter content (%) and pH were determined. The potassium dichromate method (NY 1121.6 2006) was employed to determine organic matter content in Cambisol samples [56]. Soil pH was measured using an electrode potentiometer in distillate water, in the ratio of 1 part soil to 2.5 parts of water (*w*/*v*).

#### 4.3.2. Measurement of the Total Number of Bacteria of Cambisols

The total number of bacteria of Cambisols reflects the state of reducers in the ecosystem [50]. The total number of bacteria of Cambisols was determined by the luminescence microscopy method considering the number of bacteria after staining with acridine orange dye [57]. Acridine orange is a fluorochromatic dye that binds to nucleic acids bacteria, and other cells. Under the influence of ultraviolet radiation, acridine orange stains ribonucleic acid (RNA) and single-stranded DNA in an orange color (as soil particles), double-stranded DNA in green (as bacterial cells). After incubation, the fresh soil was dried and a soil suspension (soil: water, 1: 100) was prepared. On prepared glasses (defatted and sterilized), 10 μL of soil suspension was placed, air-dried (air temperature—22–24 °C), and dried in a burner flame (duration 3–5 s). After that, the glasses were stained with a solution of acridine orange dye (dilution of the solution of acridine orange dye, 1:100,000) for 20 min. The glasses were washed to remove excess dye and dried in the air. The glasses were viewed under a Carl Zeiss Axio Lab A1 microscope at a magnification of X40 (20 bacterial cells of counting fields).

#### 4.3.3. Measurement of *Azotobacter* sp. Abundance

*Azotobacter* sp. abundance was traditionally used to indicate chemical pollution of the soils [58]. The abundance of bacteria of the genus *Azotobacter* sp. was determined by the method of fouling lumps on Ashby medium. To assess the number of bacteria, Ashby’s medium was prepared. The medium was poured into Petri dishes and lumps of moistened soil (25 pieces per 1 dish, temperature of incubation, 22–25 °C) were stirred. These operations were performed in an abacterial air-box BAVnp-01—“Laminar-S”. The number of fouling lumps was counted 14 days after the start of the experiment. Counting of soil lumps overgrown with *Azotobacter* sp. abundance was carried out relative to the control.

#### 4.3.4. Measurement of the Activity of Catalase and Dehydrogenases of Cambisols

The activity of catalase and dehydrogenases estimated the potential biological activity in soils. Oxidoreductases (catalase and dehydrogenases) were more sensitive to chemical pollution than other enzymes [59]. Catalase activity was determined according to Galstyan’s (1978) [60]. The gasometric method determined the enzyme activity by the rate of decomposition of 5% hydrogen peroxide after contact with the soil (temperature, 20–22 °C). Dehydrogenases were determined according to Galstyan’s method modified by Khaziev. The activity of dehydrogenases was determined by the conversion of TPC to TPF. The optical density of the colored solutions was determined spectrophotometrically on a PE 5800VI spectrophotometer at a wavelength of 540 nm.

#### 4.3.5. Measurement of Germination Rate and Length of Radish Roots 

Soil phytotoxicity was investigated by the germination rate of radish and length of roots in growth chamber Binder KBW 240 [61]. Compared to other plant test objects, radish had a fast response to soil nutrients and moisture [62]. Germination rate and root length of radish were the most informative of the many indicators of soil phytotoxicity [52,63].

After incubation of the Cambisols with CuNPs, NiNPs, and ZnNPs for 10 days, the soil was placed in a Petri dish. Twenty five radish seeds were planted in each Petri dish in conditions of moisture 60% and temperature of 24–25 °C. After 7 days of the experiment, the radish was pulled out of the soil and the germination rate and the length of roots were determined. Germination rate was assessed by the number of germinated seeds in 7 days of the experiment (after the appearance of 2 or more leaves).

### 4.4. Data Analysis

The results of Cambisols bacteria were expressed in 10^9^ bacteria in gram of dry soil weight. This is example 1 of an equation:

(1) $$M=\frac{b\times A\times H\times T}{P}$$

where M is the number of cells per 1 g of fresh soil; A—the average number of cells within one field of vision; b—coefficient magnification factor (b = 4); H—dilution index; T—conversion factor in billions of bacteria per 1 g of soil (T = 10^10^); and P—the area of the field of vision, µm^2^.

The indices of the intensity of the initial growth of radish seeds (length of radish roots) were calculated as the average triplicate. This is example 2 of an equation:

(2) $$G=\frac{n_{1}+\ldots +{\;n}_{m\;}}{m}$$

where G is the germination rate; n_1_—number of the seed of 1st replicate; n_m_—number of the seed of m replicate; and m—quantity of replicates.

Integrated index of biological state (IIBS) of the soil was allowed to give an integral assessment of the condition of soils after any chemical pollution [15]. For the calculation of IIBS, the value of each of the above indicators on the control (in unpolluted soil) was taken as 100%. The percentages in other experimental variants (in polluted soil) were expressed as a percentage relative to control. For the IIBS condition, the maximum value of each index (100%) was chosen from array data. This is example 3 of an equation:

(3) $$B_{1\;}=\frac{B_{x}}{B_{\max}}\times 100\%$$

where B_1_ is the relative score of the indicator; B_x_—the actual value of the indicator; and B_max_—the maximum value of the indicator.

The relative values of several mostly informative indices of soil biological condition such as the activity of catalase and dehydrogenases, total number of bacteria, *Azotobacter* sp. abundance, length of roots, and germination rate of radish seeds were summed.

Thereafter, the average assessment point of studied indices was calculated for each variant. This is example 4 of an equation:

(4) $$B=\frac{B_{1}+B_{2}+\ldots +B_{n}}{N}$$

where B is the average estimated score of indicators; B_1_…B_n_—the relative score of the indicator; and N—the number of indicators.

The integral index of the soil biological state (IIBS) is calculated. This is example 5 of an equation:

(5) $$\mathrm{IIBS}=\frac{B}{B_{\max}}\times 100\%$$

where B is the average estimated score of all indicators and B_max_ is the maximum estimated score of all indicators.

During diagnostics of the contamination value of each index in non-contaminated soil, it was taken as 100%. With reference to its value, the same index in the contaminated soil was expressed in percent, and then the average value of 6 selected biological indicators for each experiment was determined. The obtained value IIBS was expressed as a percentage concerning the control (to 100%). The used methodology allowed integrating the relative values of different biological indicators, which cannot be integrated since they have different units of measurement. Thanks to this methodology of IIBS, it is possible to compare the biological indicators of soils in terms of relative values (relative to control, 100%). The soil condition was assessed based on the change in IIBS on the scale of the change in the indicator relative to the control (%). With a change in IPBS of <5%, no significant changes in the state of the soil are recorded (no changes). If a change is found in the range of 5–10% of the control, then a change in information functions is detected, with a decrease of 10–25% of the control, a violation of the chemical, physical, and biochemical functions of the soil. When the IIBS changes by >25%, the physical functions of the soil are disturbed. In this case, the soil requires a long recovery period. The degree of sensitivity of biological indicators was assessed by the degree of decrease in the values of the biological indicator compared to the control. The more the value of the biological indicator decreased from the control (100%), the more sensitive this biological indicator was.

The informative value was assessed by the tightness of the correlation between the biological indicator and the concentration of the substance in the soil. The closer the correlation coefficient is R = −1, the higher the information content of this biological indicator was.

### 4.5. Statistical Analyses

Statistical calculations and data visualization were performed using Statistica 7 and Sigmaplot 12.5 software. The significance of the differences between the options was assessed using the Student’s t test. Regression analysis was carried out using exponential equations. Data analysis was carried out at 95% probability (at *p* < 0.05).

## 5. Conclusions

It was found that the contamination of Cambisols with nanoparticles of Cu, Zn, and Ni causes deterioration of their biological properties, such as the total number of bacteria, the *Azotobacter* sp. abundance, the activity of catalase and dehydrogenases, seed germination rate, and the length of radish roots. According to the degree of the negative influence of NPs on the biological properties of Cambisols, the studied metals formed the sequence: Cu ≥ Zn> Ni. A direct dependence of the deterioration of the biological properties of the soil on the concentration of metal-based NPs was observed. The stimulating effects of CuNPs, ZnNPs, and NiNPs were not revealed, which is often observed when the soil is contaminated with metal based NPs or heavy metals. The microbiological and phytotoxic properties of the Cambisols deteriorate to the greatest extent, and the enzymatic activity is less sensitive to contamination with Cu, Zn, Ni NPs. The studies on the alteration of the biological status of Cambisol from the contamination with NPs of Cu, Zn, and Ni show that the ecotoxicity assessment of these NPs are essential and advisable to conduct a series of study to understand the ecotoxicity in soil by focusing on NPs of various sizes and oxide forms of metals. The results could enhance the understanding for biomonitoring and biodiagnostics of the state of soils and agricultural plants after NP contamination.

## Figures and Tables

**Table 1:** Characteristics of biological indicators of Cambisols’ condition.

No	Biological Indicators	Measure Unit	Methods
1	total number of bacteria	10^9^ bacteria in gram of dry soil weight	luminescent microscopy with the solution of acridine orange, 40×
2	*Azotobacter* sp. abundance	% of the mud balls surrounded by Azotobacter mucus	the method of fouling lumps on the Ashby medium
3	catalase activity	ml O_2_ per gram of soil dry weight in 1 min.	by the rate of decomposition of hydrogen peroxide
4	dehydrogenases activity	mg of triphenylformazane per gram of dry soil weight for hour	according to the rate of conversion of triphenyltetrazolium chloride (TPC) to triphenylformazane (TPF)
5	the germination rate of radish seeds	% of germination seeds of control	germination of radish (*Raphanus sativus* L.) after 7 days of the experiment
6	the length of the radish roots	millimeters	of length of the roots in radish (*Raphanus sativus* L.) after 7 days of the experiment

## Data Availability

The data presented in this study are available upon request from the respective author.

## References

[1] Adams M.B., Kelly C., Kabrick J., Schuler J. (2019). Chapter 6—Temperate forests and soils. Dev. Soil Sci..

[2] IUSS Working Group WRB (2015). World Reference Base for Soil Resources 2014, update 2015. International soil classification system for naming soils and creating legends for soil maps.

[3] Nafisi S., Maibach H.I. (2017). Nanotechnology in cosmetics. Cosmet. Sci.Technol. Theor. Princ. Appl..

[4] (2020). The National Nanotechnology Initiative Supplement to the President’s 2021 Budget. https://www.nano.gov/nanodashboard.

[5] (2020). Global Nanotechnology Market Outlook 2020–2025 Segmented by Type, Applications & End-User Industries, Business Wire.

[6] Abbas Q., Yousaf B., Ali A.M.U., Munir M.A.M., El-Naggar A., Rinklebe J., Naushad M. (2020). Transformation pathways and fate of engineered nanoparticles (ENPs) in distinct interactive environmental compartments: A review. Environ. Int..

[7] Ahmed B., Khan M.S., Musarrat J. (2018). Toxicity assessment of metal oxide nano-pollutants on tomato (*Solanum lycopersicon*): A study on growth dynamics and plant cell death. Environ. Pollut..

[8] Lead J.R., Batley G.E., Alvarez P.J.J., Croteau M.N., Handy R.D., McLaughlin M.J., Judy J.D., Schirmer K. (2018). Nanomaterials in the environment: Behavior, fate, bioavailability, and effects—An updated review. Environ. Toxicol. Chem..

[9] Rajput V.D., Minkina T., Fedorenko A., Mandzhieva S., Sushkova S., Lysenko N.D.V., Azarov A., Chokheli V. (2019). Destructive effect of copper oxide nanoparticles on ultrastructure of chloroplast, plastoglobules and starch grains in spring barley (*Hordeum sativum*). Int. J. Agric. Biol..

[10] Zhang P., Guo Z., Zhang Z., Fu H., White J.C., Lynch I. (2020). Nanomaterial transformation in the soil-plant system: Implications for food safety and application in agriculture. Small.

[11] Abd-Elsalam K.A. (2021). Zinc-Based Nanostructures for Environmental and Agricultural Applications.

[12] Sanzari I., Leone A., Ambrosone A. (2019). Nanotechnology in plant science: To make a long story short. Front. Bioeng. Biotechnol..

[13] Soltanian S., Sheikhbahaei M., Mohamadi N., Pabarja A., Abadi M.F.S., Tahroudi M.H.M. (2021). Biosynthesis of Zinc Oxide Nanoparticles Using Hertia intermedia and Evaluation of its Cytotoxic and Antimicrobial Activities. BioNanoScience.

[14] Rajput V., Minkina T., Fedorenko A., Sushkova S., Mandzhieva S., Lysenko V., Duplii N., Fedorenko G., Dvadnenko K., Ghazaryan K. (2018). Toxicity of copper oxide nanoparticles on spring barley (*Hordeum sativum* distichum). Sci. Total Environ..

[15] Kolesnikov S.I., Kazeev K.S., Akimenko Y.V. (2019). Development of regional standards for pollutants in the soil using biological parameters. Environ. Monit. Assess..

[16] Xu C., Peng C., Sun L., Zhang S., Huang H., Chen Y., Shi J. (2015). Distinctive effects of TiO_2_ and CuO nanoparticles on soil microbes and their community structures in flooded paddy soil. Soil Biol. Biochem..

[17] Ameen K.I., Alabdullatif J.A., AL-Nadhari S. (2021). A review on metal-based nanoparticles and their toxicity to beneficial soil bacteria and fungi. Ecotoxicol. Environ. Saf..

[18] Khan S.A., Shahid S., Ayaz A., Alkahtani J., Elshikh M.S., Riaz T. (2021). Phytomolecules-Coated NiO Nanoparticles Synthesis Using *Abutilon indicum* Leaf Extract: Antioxidant, Antibacterial, and Anticancer Activities. Nanomedicine.

[19] Adams J., Wright M., Wagner H., Valiente J., Britt D., Anderson A. (2017). Cu from dissolution of CuO nanoparticles signals changes in root morphology. Plant Physiol. Biochem..

[20] AlQuraidi A.O., Mosa K.A., Ramamoorthy K. (2019). Phytotoxic and Genotoxic Effects of Copper Nanoparticles in Coriander (*Coriandrum sativum*—Apiaceae). Plants.

[21] Tanha E.Y., Fallah S., Rostamnejadi A., Lok P. (2020). Particle size and concentration dependent toxicity of copper oxide nanoparticles (CuONPs) on seed yield and antioxidant defense system in soil grown soybean (Glycine max cv. Kowsar). Sci. Total. Environ..

[22] Tsitsuashvili V.S., Minkina T.M., Nevidomskaya D.G., Rajput V.D., Mandzhieva S.S., Sushkova S.N., Bauer T.V., Burachevskaya M.V. (2017). The impact of copper nanoparticles on plants and soil microorganisms (literature review). Bull. Agrar. Sci. Don.

[23] Korotkova A.M., Lebedev S.V., Kayumov F.G., Sizova E.A. (2017). The influence metal nanoparticles (Fe, Cu, Ni) and their oxides (Fe_3_O_4_, CuO, NiO). Sel’skokhozyaistvennaya Biol..

[24] Zotikova A.P., Astafurova T.P., Burenin A.A., Suchkova S.A., Morgalev Y.N. (2018). Morphophysiological features of wheat seedlings (*Triticum Aestivum* L.) When exposed to nickel nanoparticles. Agric. Biol..

[25] Ghosh M., Jana A., Sinha S., Jothiramajayam M., Nag A., Chakraborty A., Mukherjee A. (2016). Effects of ZnO nanoparticles on plants: Cytotoxicity, genotoxicity, deregulation of antioxidant defenses, and cell-cycle arrest. Mutat. Res. Genet. Toxicol. Environ. Mutagen.

[26] Shen M., Liu W., Zeb A., Lian J., Hu X., Wu J. (2021). Bioaccumulation and Phytotoxicity of ZnO Nanoparticles in Soil-Grown Brassica chinensis and Potential Risks. https://assets.researchsquare.com/files/rs-202101/v1/f3a5e4c2-da2d-4644-aebf-a659cd0475ad.pdf?c=1631876197.

[27] Zoufan P., Baroonian M., Zargar B. (2020). ZnO nanoparticles-induced oxidative stress in Chenopodium murale L, Zn uptake, and accumulation under hydroponic culture. Environ. Sci. Pollut. Res..

[28] Josko I., Oleszczuk P., Dobrzyńska J., Futa B., Joniec J., Dobrowolski R. (2019). Long-term effect of ZnO and CuO nanoparticles on soil microbial community in different types of soil. Geoderma.

[29] Kim S., Sin H., Lee S., Lee I. (2013). Influence of Metal Oxide Particles on Soil Enzyme Activity and Bioaccumulation of Two Plants. J. Microbiol. Biotechnol..

[30] Zhao S., Su X., Wang Y., Yang X., Bi M., He Q., Chen Y. (2020). Copper oxide nanoparticles inhibited denitrifying enzymes and electron transport system activities to influence soil denitrification and N_2_O emission. Chemosphere.

[31] Avila-Arias H., Nies L.F., Gray M.B., Turco R.F. (2019). Impacts of molybdenum-, nickel-, and lithium-oxide nanomaterials on soil activity and microbial community structure. Sci. Total Environ..

[32] Galaktionova L., Gavrish I., Lebedev S. (2019). Bioeffects of Zn and Cu Nanoparticles in Soil Systems. Toxicol. Environ. Health Sci..

[33] Kumar A., Rakshit R., Bhowmik A., Mandal N., Das A., Adhikary S. (2019). Tumor-Targeting NIRF NanoGUMBOS with Cyclodextrin-Enhanced Chemo. Photothermal Antitumor Act. ACS Appl. Mater. Interfaces.

[34] You T., Liu D., Chen J., Yang Z., Dou R., Gao X., Wang L. (2018). Effects of metal oxide nanoparticles on soil enzyme activities and bacterial communities in two different soil types. J. Soils Sediments.

[35] Ali S., Rizwan M., Noureen S., Anwar S., Ali B., Naveed M., Abd Allah E.F., Alqarawi A.A., Ahmad P. (2019). Combined use of biochar and zinc oxide nanoparticle foliar spray improved the plant growth and decreased the cadmium accumulation in rice (*Oryza sativa* L.) plant. Environ. Sci. Pollut. Res..

[36] Ananda S., Shobha G., Shashidhara K.S., Mahadimane V. (2019). Nano-cuprous oxide enhances seed germination and seedling growth in Lycopersicum esculentum plants. J. Drug Deliv. Ther..

[37] Bashir A., Rizwan M., Ali S., Adrees M., Rehman M.Z., UrQayyum M.F. (2020). Effect of composted organic amendments and zinc oxide nanoparticles on growth and cadmium accumulation by wheat; a life cycle study. Environ. Sci. Pollut. Res..

[38] Dimkpa C.O., Singh U., Bindraban P.S., Elmer W.H., Gardea-Torresdey J.L., White J.C. (2019). Zinc oxide nanoparticles alleviate drought-induced alterations in sorghum performance, nutrient acquisition, and grain fortification. Sci. Total. Environ..

[39] Luying S., Fengbin S., Xiangnan L., Xiancan Z., Shengqun L., Yang W., Xiaoning Q. (2020). Effects of ZnO nanoparticles on seed germination and root carbon metabolism in maize (*Zea mays* L.). Soils Crop.

[40] Ma X., Sharifan H., Dou F., Sun W. (2020). Simultaneous reduction of arsenic (As) and cadmium (Cd) accumulation in rice by zinc oxide nanoparticles. Chem. Eng. J..

[41] Singh R.P., Handa R., Manchanda G. (2021). Nanoparticles in sustainable agriculture: An emerging opportunity. J. Control. Release.

[42] Venzhik Y.V., Moshkov I.E., Dykman L.A. (2021). Influence of nanoparticles of metals and their oxides on the photosynthetic apparatus of plants. Biol. Bulliten Russ. Acad. Sci..

[43] Chang H., Jwo C., Lo C., Tsung T., Kao M., Lin H.-M. (2004). Rheology of CuO nanoparticle suspension prepared by ASNSS. Rev. Adv. Master Sci..

[44] Osmond-McLeod M., McCall M. (2010). Zinc oxide nanoparticles in modern sunscreens: An analysis of potential exposure and hazard. Nanotoxicology.

[45] Wang W., Ren Y., He J., Zhang L., Wang X., Cui Z. (2020). Impact of copper oxide nanoparticles on the germination, seedling growth, and physiological responses in *Brassica pekinensis* L.. Environ. Sci. Pollut. Res..

[46] Kolesnikov S., Tsepina N., Minnikova T., Kazeev K., Mandzhieva S., Sushkova S., Minkina T., Mazarji M., Singh R.K., Rajput V.D. (2021). Influence of Silver Nanoparticles on the Biological Indicators of Haplic Chernozem. Plants.

[47] Gautama A., Raya A., Mukherjeea S., Dasa S., Palb K., Dasc S., Karmakar P., Ray M., Ray S. (2018). Immunotoxicity of copper nanoparticle and copper sulfate in a common Indian earthworm. Ecotoxicol. Environ. Saf..

[48] Gomes S.I.L., Murphy M., Nielsen M.T., Kristiansen S.M., Amorim M.J.B., Scott-Fordsmand J.J. (2015). Cu-nanoparticles ecotoxicity-explored and explained?. Chemosphere.

[49] Joskoa I., Oleszczukb P., Futa B. (2014). The effect of inorganic nanoparticles (ZnO, Cr_2_O_3_, CuO and Ni) and their bulk counterparts on enzyme activities in different soils. Geoderma.

[50] Kolesnikov S.I., Timoshenko A.N., Kazeev K.S., Akimenko Y.V., Myasnikova M.A. (2019). Ecotoxicity of copper, nickel, and zinc nanoparticles assessment on the basis of biological indicators of chernozems. Eurasian Soil Sci..

[51] Varduni V.M., Kolesnikov S.I., Timoshenko A.N., Kazeev K.S., Akimenko Y.V. (2019). Influence of AL_2_O_3_, TIO_2_, Fe_2_O_3_ and SIO_2_ nanoparticles on the biological state of ordinary chernozem. North Cauc. Region. Ser. Nat. Sci..

[52] Kolesnikov S.I., Kazeev K.S., Valkov V.F. (1999). The Effect of Heavy Metal Contamination on the Microbial System in Chernozem. Eurasian Soil Sci..

[53] Paz-Ferreiro J., Baez-Bernal D., Castro Insúa J., García M.I. (2011). Pomar Effects of mussel shell addition on the chemical and biological properties of a Cambisol. Chemosphere.

[54] Zakarauskaitė D., Vaišvila Z., Motuzas A., Grigaliūnienė K., Buivydaitė V., Vaisvalavičius R., Butkus V. (2008). The influence of long-term application of mineral fertilizers on the biological activity of Cambisols. Ekologija.

[55] Kabata-Pendias A. (2010). Trace Elements in Soils and Plants.

[56] Wylie E.M., Colletti L.M., Walker L.F., Lujan E.J., Garduno K., Mathew K.J. (2018). Comparison of the Davies and Gray titrimetric method with potassium dichromate and ceric titrants. J. Radioanalitical Nucl. Chem..

[57] McFeters G.A., Yu F.P., Pyle B.H., Stewart P.S. (1995). Physiological assessment of bacteria using fluorochromes. J. Microbiol. Methods.

[58] Val’kov V.F., Kolesnikov S.I., Kazeev K.S., Tashchiev S.S. (1997). Influence of heavy metal pollution on microscopic fungi and Azotobacter of common chernozem. Russ. J. Ecol..

[59] Martinez M., Gutiérrez-Romero V., Jannsens M., Ortega-Blu R. (2010). Biological soil quality indicators: A review. Curr. Res. Technol. Educ. Top. Appl. Microbiol. Microb. Biotechnol..

[60] Galstyan A.S. (1978). Unification of methods for studying the activity of soil enzymes. Eurasian Soil Sci..

[61] Bab’eva M.A., Zenova N.K. (1989). Soil Biology.

[62] Pandey S.N. (2006). Accumulation heavy metals (cadmium, cromium, copper, nickel and zinc) in *Raphanus salivus* L. and *Spinacia olerac* L. Plants Irrigated with Industrial Effluents. J. Environ. Biol..

[63] Plekhanova I.O., Zolotareva O.A., Tarasenko I.D., Yakovlev A.S. (2019). Assessment of ecotoxicity of soils contaminated by heavy metals. Eurasian Soil Sci..

